# Exploring predictors of insomnia severity in shift workers using machine learning model

**DOI:** 10.3389/fpubh.2025.1494583

**Published:** 2025-03-14

**Authors:** Hyewon Yeo, Hyeyeon Jang, Nambeom Kim, Sehyun Jeon, Yunjee Hwang, Chang-Ki Kang, Seog Ju Kim

**Affiliations:** ^1^Samsung Medical Center, Sungkyunkwan University, Seoul, Republic of Korea; ^2^Medical Campus, Biomedical Engineering Research Center, Gachon University, Incheon, Republic of Korea; ^3^Brain and Cognitive Engineering, Korea University, Seoul, Republic of Korea; ^4^Medical Campus, Health Science, Radiological Science, Gachon University, Incheon, Republic of Korea; ^5^School of Medicine, Psychiatry, Sungkyunkwan University, Suwon, Republic of Korea

**Keywords:** shift worker, sleep, insomnia, machine learning, risk prediction

## Abstract

**Introduction:**

Insomnia in shift workers has distinctive features due to circadian rhythm disruption caused by reversed or unstable sleep-wake cycle work schedules. While previous studies have primarily focused on a limited number of predictors for insomnia severity in shift workers, there is a need to further explore key predictors, and develop a data-driven prediction model for insomnia in shift workers. This study aims to identify potential predictors of insomnia severity in shift workers using a machine learning (ML) approach and evaluate the accuracy of the resulting prediction model.

**Methods:**

We assessed the predictors of insomnia severity in large samples of individuals (4,572 shift workers and 2,093 non-shift workers). The general linear model with the least absolute shrinkage and selection operator (LASSO) was used to determine an ML-based prediction model. Additional analyses were conducted to assess the interaction effects depending on the shift work schedule.

**Results:**

The ML algorithms identified 41 key predictors from 281 variables: 1 demographic, 7 physical health, 13 job characteristics, and 20 mental health factors. Compared to the non-shift workers, the shift workers showed a stronger association between insomnia severity and five predicting variables: passiveness at work, authoritarian work atmosphere, easiness to wake up, family and interpersonal stress, and medication. The prediction model demonstrated good performance with high accuracy and specificity overall despite a limited F1 score (classification effectiveness) and recall (sensitivity). Specifically, a prediction model for shift workers showed better balance in F1 scores and recall compared to that for non-shift workers.

**Discussion:**

This ML algorithm provides an effective method for identifying key factors that predict insomnia severity in shift workers. Our findings align with the traditional insomnia model while also reflecting the distinctive features of shift work such as workplace conditions. Although the potential for immediate clinical application is limited, this study can serve as guidance for future research in improving a prediction model for shift workers. Constructing comprehensive ML-based prediction models that include our key predictors could be a crucial approach for clinical purposes.

## Highlights

- Machine learning enhanced the accuracy and efficiency of the prediction model.
- Our model predicted the severity of insomnia in shift workers.
- Anxiety-related predictors aligned with the traditional hyperarousal model of insomnia.
- Work-related predictors represented distinctive risk factors for insomnia in shift workers.

## Background

Insomnia is a complex psychosomatic condition that is defined as a non-restorative sleep with the repeated occurrence of increased sleep latency, difficulty in staying asleep, and early waking despite favorable sleeping opportunities. Within a diathesis-stress model, predisposing factors (e.g., biological predisposition and psychological vulnerabilities) interacting with precipitating factors (e.g., environmental stressors, sleep hygiene, substance abuse, and poor socioeconomic background) trigger the development of insomnia (1, 2). Demographic factors, such as gender, age, and familial history of insomnia, contribute to the risk of insomnia (3). Conversely, behavioral factors (e.g., alcohol use) and psychological traits (e.g., anxiety-prone personality, worries, social introversion, and poor coping ability) (4, 5) are also implicated in the development of insomnia. Furthermore, mental health problems, such as anxiety and mood (6, 7), and physical health conditions, such as heart disease and urinary problems (5, 8), have been identified as significant risk factors for insomnia.

Previous studies have focused on these variables to find prediction models, and multivariate models have attempted to incorporate as many possible variables as possible to enhance the accuracy. However, while improving the predictive power, including too many variables compromises the practical aspects, such as clinical explainability. Since it is costly to include all variables in treatment plans in the clinical setting, selecting a few key variables in prediction models is important to enhance the treatment efficacy. The introduction of ML, a cutting-edge analysis tool for big datasets, presents a promising solution to these challenges. The prediction power of ML outperforms that of traditional models in handling numerous variables. Moreover, ML techniques such as feature selection enable the effective reduction of variables by identifying the key predictors based on their predictive power.

Recent studies have applied ML techniques, such as logistic regression (LR), XGBoost, random forest, and artificial neural network (ANN), for the prediction of insomnia (9, 10). A comparative study of 15 ML algorithms has found that insomnia primarily depended on the following factors: sleep disorder, vision problems, and mobility problems (11). Although those machine learning models have evaluated potential predictors in sleep disorders, no study has specifically focused on insomnia in shift workers. This gap may be critical, as there are both common and distinct factors in insomnia among shift workers compared to the general population.

The traditional hyperarousal model of insomnia suggests that hyperarousal in cognitive, emotional, cortical, and physiological domains precipitates and perpetuates insomnia (12–14). Hyperarousal refers to a heightened state of arousal across those domains, manifesting both during the day and night in individuals with insomnia disorder. Previous studies have attributed hyperarousal in insomnia disorder to cognitive-behavioral dysfunctions, including conditioned arousal to the bed environment, excessive sleep effort, and dysfunctional beliefs about sleep (15–17), along with neural dysregulations in sleep–wake circuits (18, 19). Hyperarousal is influenced by psychiatric traits, such as neuroticism, which predicts both shift work tolerance and insomnia symptoms among shift workers (20, 21). High sleep reactivity, which represents the enhanced hyperarousal response to stressors, was also reported in shift workers (22). This indicates that the hyperarousal model of insomnia may not only be relevant to shift workers but could also manifest more strongly in them, as circadian rhythm misalignment in shift work might be linked to heightened sleep reactivity, potentially contributing to greater hyperarousal and more severe insomnia.

Insomnia in shift workers differs significantly from that experienced by non-shift workers. Shift workers, who alternate between day and night shifts, are at risk of developing shift work disorder (SWD), a circadian rhythm sleep–wake disorder. Unlike general insomnia, SWD is characterized not only by insomnia during the desired sleep period but also by excessive sleepiness during the desired waking period. According to the definition of SWD, these symptoms are not explained by other sleep disorders, medical conditions, or medication effects, indicating that environmental factors have significant impacts on the onset and recovery of SWD. It would be necessary to investigate the insomnia of shift workers separately from that of non-shift workers, due to the unique symptoms of SWD. For example, excessive sleepiness is more common in SWD compared to general insomnia in non-shift workers (23). Excessive sleepiness is strongly related to circadian rhythm misalignment, while general insomnia is more associated with physiological hyperarousal (24). In addition, even under the same shift work environment, some workers develop insomnia while others do not. Though environmental factors such as work schedules are important for the development of SWD, individual traits such as stress tolerance and circadian preferences can also influence SWD (25). Thus, a wide range of potential predictors encompassing environmental and individual factors would be needed for understanding the insomnia of shift workers.

The primary goal of this study is to identify potential predictors of insomnia severity in shift workers using an ML approach. The secondary goal is to derive the optimal machine learning algorithm for predicting insomnia severity among shift workers and evaluate the accuracy of the resulting prediction models. Addressing limitations of previous research, such as the lack of large-scale data and insufficient focus on shift work, this study incorporates all potential variables associated with SWD into the ML model. Given the exploratory nature of the study, no specific hypotheses were formulated regarding the effects of different variables on insomnia severity.

## Materials and methods

### Recruitment

The initial recruitment used both online and offline bulletin boards of Samsung Medical Center. The study participants were encouraged to introduce the recruitment to other potential participants, especially those who were shift workers. The initially recruited participants had various professions, including healthcare workers, police officers, drivers, firefighters, and factory workers. The proportion of young female shift workers was larger among the initially recruited 1,254 participants (448 men and 806 women; 961 shift workers and 293 non-shift workers; 32.6 ± 7.9 years old). To add more male participants, middle-aged workers, and non-shift workers, an online research company (Macromill Embrain Co., Ltd.) recruited an additional 5,400 participants (2,693 men and 2,707 women; 3,600 shift workers and 1,800 non-shift workers; 38.3 ± 9.9 years old). The online survey company has its own research panel consisting of more than 1 million individuals. These individuals either voluntarily joined the panel or were recruited through referrals from existing panel members. Finally, the present study enrolled a total of 6,665 subjects (4,572 shift workers and 2,093 non-shift workers).

All participants completed online surveys using the KakaoTalk service. KakaoTalk is the largest social networking service in the Republic of Korea, primarily used as a messaging app for communication and business interactions. Up to 97.5% of mobile messaging app users in South Korea use KakaoTalk (26), which allowed participants to conveniently engage in the online survey. To address potential privacy concerns, data is thoroughly secured following the IRB ethics protocols at Samsung Medical Center.

Participants aged at least 18 years and working full or part-time were included, whereas those who did not complete the online surveys were excluded. The enrolled participants were then divided into two groups. Based on the classification of previous studies (27), the present study has defined the shift workers. Non-shift workers were those who worked regular and fixed schedules during the daytime from 7 AM to 6 PM. Shift workers include those with evening, night, rotating, casual, or flexible schedules. These diverse types of shift workers, whose hours fall outside regular working times, were categorized as the “shift worker” group. Following are the explanations for each shift work type: Fixed evening shift workers typically work from 3 PM to 11 PM, while fixed night shift workers work from 11 PM to 7 AM. Regular rotating shift workers follow predictable, regularly changing shifts, whereas irregularly rotating shift workers experience irregular but still predictable changes in their schedules. Casual shift workers, such as actors or photography directors, have unpredictable schedules often involving nighttime work. The part-time workers are different from casual workers. The part-time or full-time work can be divided by working hours rather than working schedules. The casual workers are defined by the work schedule, which is irregular, non-predictable, and cannot be changed at the will of workers. Flexible shift workers determine their work time based on individual preference, but often at night. Participants who did not fit into any of these categories were classified as Others. In total, the present study included 4,572 shift workers [37.0 ± 9.84 years of age, 2,150 males (47.03%)] and 2,093 non-shift workers [37.8 ± 9.73 years of age, 999 males (47.73%)].

The study procedures followed the ethical standards of the relevant institutional committees on human experimentation and the Declaration of Helsinki (2013). The study protocol was approved by the Institutional Review Board of Samsung Medical Center (protocol code: 2019-04-095). All participants provided informed consent.

### Potential predictors of insomnia severity

In the present study, potential predictors of insomnia severity were selected and put into the ML algorithm as input variables. Among the 630 variables in the initial online survey, 278 were excluded because they were either overlapping items already included in the output variable (i.e., sleep duration, daytime symptoms) or were considered consequences of insomnia rather than potential predictors (i.e., sleep-related mood symptoms). Specifically, daytime symptoms criteria of insomnia disorders presented in International Classification of Sleep Disorder-3 were considered as consequences of insomnia. By excluding overlapping items which would have introduced redundancy and potentially biased the results, we focused on identifying true predictors and enhancing the validity of the model’s predictions. Moreover, 64 variables were also excluded for the following reasons: 59 variables which can be answered by only part of the participants (e.g., “If you are a smoker, how much do you smoke a day?”), 4 unquantifiable variables (e.g., “What is the name of your workplace?”), and 1 variable duplicated with other variables (e.g., body mass index, which can be calculated by height and weight). Finally, 281 variables were selected as the potential predictors of insomnia severity and applied to the ML algorithm. Detailed explanation about potential predictors were demonstrated in the Supplementary Table 1.

Of the 281 variables selected as potential predictors of insomnia, 7 items assessed the demographics, 96 items assessed the job characteristics, 25 items assessed physical health, and 153 items assessed mental health (Table 1). Among the 281 variables, 217 items (58 job characteristics items, 6 physical health items, and 153 mental health items) were selected from pre-existing validated questionnaires, while the other 64 items (7 demographic items, 38 job characteristics items, and 19 physical health items) were designed after discussions with three mental health professionals.

**Table 1:** Potential predictors of insomnia severity.

Categories	Number of items	Potential predictors of insomnia severity
Demographics	7	Gender (1 item), age (1 item), education (1 item), family (4 items)
Job Characteristics	96	Work schedule (25 items), time-zone shift (1 item), working period (3 items), working hours per week (1 item), sideline (1 item), commute (3 items), self-reported workload (4 items), Maslach Burnout Inventory-General Survey (15 items), Korean Occupational Stress Scale (43 items)
Physical Health	25	Height/weight (3 items), alcohol/smoking/coffee (5 items), current/past medical disease (2 items), medication (1 item), family history of sleep disorder (1 item), eating/diet (4 items), exercise (3 items), Berline Questionnaire (5 items^a^), International Restless Legs Scale (1 item)
Mental Health	153	Big Five Inventory (10 items), Stress Vulnerability Scale (20 items), Global Assessment of Recent Stress (8 items), Brief Resilience Scale (6 items), Attention-Deficit/Hyperactivity Disorder Scale (5 items), short form of the UPPS-P Impulsive Behavior Scale (20 items), World Health Organization Quality of Life Brief Version (26 items), Rothwell and Cohen’s Happiness Scale (4 items), State–Trait Anxiety Inventory-Trait (20 items), Mood Disorder Questionnaire (15 items), Morningness–Eveningness Questionnaire (19 items)

a Items directly related to insomnia were excluded.

Two questionnaires were used to assess the job characteristics: the Maslach Burnout Inventory-General Survey (MBI-GS) (28, 29) for assessing job burnout (15 items) and the Korean Occupational Stress Scale (KOSS) (30) for assessing workplace environment (43 items). Moreover, two questionnaires were used to assess physical health: the Berlin Questionnaire (BQ) (31, 32) for assessing sleep-related breathing disorders (5 items) and the International Restless Legs Scale (IRLS) (33, 34) for assessing restless legs syndrome (RLS) (1 item). Among the items of the BQ, the questions directly related to insomnia were excluded. Only one item of the IRLS for screening was used, as other questions can be asked only when RLS symptoms exist.

Eleven questionnaires were used to assess mental health: the Big Five Inventory (BFI-10) (35, 36) for assessing personality traits (10 items), the Stress Vulnerability Scale (SVS) (37) for assessing vulnerability to stress (20 items), the Global Assessment of Recent Stress (GARS) (38, 39) for assessing stressful experiences (8 items), the Brief Resilience Scale (BRS) (40, 41) for measuring resilience (6 items), the Adult Attention-Deficit/Hyperactivity Disorder Rating Scale for assessing inattentiveness and hyperactivity (5 items) (42, 43), the short form of the UPPS-P Impulsive Behavior Scale (SUPPS-P) (44, 45) for assessing impulsivity (20 items), the World Health Organization Quality of Life Brief Version (WHOQOL-BREF) for assessing quality of life (26 items) (46, 47), the State–Trait Anxiety Inventory-Trait (STAI-T) (48, 49) for assessing trait anxiety (20 items), the Rothwell and Cohen’s happiness formula (50) for assessing happiness (4 items), the Mood Disorder Questionnaire (MDQ) (51, 52) for assessing mood bipolarity (15 items), and Morningness–Eveningness Questionnaire (MEQ) (53, 54) for assessing circadian preferences (19 items).

### Output data

The total score of the Insomnia Severity Index (ISI) was used as the output data. The ISI is a self-reported measure designed to assess the perceived severity of insomnia over a two-week recall period, using 7 items. The ISI can be used as either a continuous measure of insomnia symptoms or as a categorical measure of clinically significant insomnia. In this study, the ISI score was used as a continuous measure of insomnia severity, with higher scores representing severe insomnia symptoms (55). Also, insomnia severity is categorized as follows: scores of 8–14 correspond to mild insomnia, 15–21 to moderate insomnia, and 22–28 to severe insomnia, based on the ISI cutoff scores (56).

### Statistical analysis

#### ML analyses

The general linear model was used for constructing the regression model. A data matrix with n × p dimension was constructed, where n is the number of participants (n = 6,665) and p is the number of features (*p* = 283). This matrix used the following preprocessing. The feature was first eliminated if more than 10% of the data had missing values (e.g., 5 features were dropped). Moreover, data having missing values were also eliminated (e.g., 372 participants were dropped). Although excluding variables with missing data may lead to potential biases due to a loss of valuable information, we chose the exclusion approach to preserve the integrity of the LASSO model. As our model is sensitive to the quality of the input data, this helps reduce imputation bias, such as artificial relationships or distortion of the data’s inherent structure. By doing so, we tried to maintain both the model’s validity and performance.

To remove the near-constant features, zero variance elimination was applied using the caret package (57) (e.g., no zero variance features). Feature selection was performed using LASSO regression to reduce the risk of overfitting and enable the use of an appropriate number of features. LASSO regression is particularly valuable when working with real-world data, where many potential predictors exist, but not all contribute meaningfully to the outcome. By shrinking less important predictors to zero, LASSO enhances the model’s robustness, improving its generalizability. In this study, LASSO was appropriate for identifying a small set of key predictors of insomnia from a large pool of variables. Linear regression with the LASSO was applied using the glmnet package (58). Specifically, LASSO limits the number of effective features via the regularization of the L1 norm and allows the selection of the most significant features. *λ* limits model overfitting by influencing the degree of shrinkage of the model parameters. For example, large *λ* forces the small coefficient to be zero. One hundred linear *λ* sequence values on the log scale ranging from λ min to λ max were searched, where λ max was set to a value at which all coefficients were zero, while λ min was set to a value at which the ratio of the smallest value to λ max was 0.01. The best regularization parameter, λ, was chosen as the value with a minimum mean squared error as a metric of model fit. A tenfold cross-validation was conducted to evaluate model performance. The split ratio between the learning and validation set was 7:3. The significant feature was considered if more than 5 times were chosen in the tenfold cross-validation. All these processes were performed in shift workers, non-shift workers, and shift + non-shift workers, separately. All analyses were conducted using the R statistical software.

For performance metrics, five indices were used: accuracy, recall, precision, specificity, and F1. Accuracy is defined as the ratio of correctly predicted examples to the total examples, offering a general measure of the model’s overall correctness. Recall (i.e., sensitivity) is the ratio of correctly predicted positive examples to the total positive examples, indicating the model’s ability to capture true positives. Precision (i.e., positive predictive value) measures the proportion of correct positive predictions out of all positive predictions, reflecting the model’s reliability in identifying true positives. Specificity, the ratio of correctly predicted negative examples to the total negative examples, evaluates the model’s capacity to avoid false positives. The F1 score, calculated as the harmonic mean of precision and recall, provides a balanced measure of a model’s performance, particularly useful in cases of imbalanced datasets where optimizing for both false positives and false negatives is critical. These metrics collectively provide a comprehensive evaluation of the model’s performance across different dimensions.

#### Other statistical analyses

The between-group differences in continuous variables were evaluated using the independent t-test. Differences involving categorical variables were analyzed using a chi-squared test. For predictors of insomnia severity only in shift workers (but not in non-shift workers) or only in non-shift workers (but not in shift workers) in the ML model, the generalized linear model was used to evaluate the interaction effects between predictors and work type (shift or non-shift work) on the dependent variable (ISI total score). Continuous predicting variables were centered in order to reduce potential problems related to high multicollinearity as well as the interpretation of the coefficient in the interaction (59). Only predictors that were significantly correlated with the ISI scores at a *p*-value of <0.05 in both shift and non-shift workers were interpreted. To provide a visual summary of the significant moderations, the association between the predictors and ISI total score was assessed, with two separate lines for non-shift workers and shift workers. All statistical analyses were performed using SPSS, version 22.

## Results

Table 2 shows the demographic and clinical characteristics of the participants. Among shift workers, approximately 45% were employed in regular rotating shift work followed by irregular rotating shift work (29.1%). In the group comparison, the proportion of gender did not significantly differ but shift workers were significantly younger than non-shift workers (*t* = 3.12, *p* = 0.002). Although over 60% of workers in each group reported no morningness-eveningness preference, the proportion of evening type was much higher among shift workers than non-shift workers (*χ*^2^ = 81.57, *p* < 0.001). The mean PSQI, ESS, and ISI were significantly higher in the shift workers than non-shift workers. (PSQI; *t* = −12.70, *p* < 0.001; ISI; *t* = −13.58, *p* < 0.001; ESS; *t* = −5.17, *p* < 0.001).

**Table 2:** Demographic and clinical characteristics of the study participants.

	Shift Workers (*n* = 4,572)	Non-Shift Workers (*n* = 2,093)	*t*, chi-square	*p*-value
Age	37.0 ± 9.84	37.8 ± 9.73	3.12	0.002^**^
Gender				
Male	2,150 (47.0%)	999 (47.7%)	0.29	0.593
Female	2,422 (53.0%)	1,094 (52.3%)		
Work patterns				
Non-shift work		2,093 (100.0%)		
Fixed evening shift work	212 (4.6%)			
Fixed night shift work	163 (3.6%)			
Regular rotating shift work	2,040 (44.6%)			
Irregular rotating shift work	1,330 (29.1%)			
Flexible shift work	363 (7.9%)			
Casual shift work	453 (9.9%)			
Others	11 (0.2%)			
Sleep preference^a^			81.57	<0.001^***^
Morning Type	208 (4.5%)	160 (7.6%)		
Evening Type	1,539 (33.7%)	497 (23.7%)		
Neither	2,825 (61.8%)	1,436 (68.6%)		
Sleep Questionnaires				
PSQI	7.4 ± 3.59	6.3 ± 3.23	−12.70	<0.001^***^
ISI	10.2 ± 6.2	8.0 ± 5.82	−13.58	<0.001^***^
ESS	8.4 ± 4.00	7.8 ± 3.88	−5.17	<0.001^***^

^**^p < 0.01, ^***^p < 0.001 by independent *t*-test or the chi-square test. PSQI, Pittsburg Sleep Quality Index; ESS, Epworth Sleepiness Scale, ISI, Insomnia Severity Scale, MEQ, Morningness-Eveningness Questionnaire.

a An MEQ score over 59 indicates morning type, a score below 41 indicates evening type, and a score between 42 and 58 indicates neither type.

### Predictors of insomnia among all participants

Among the 281 input variables, the ML algorithm selected following 46 variables for predicting insomnia among all participants (Table 3): 1 demographic (i.e., co-sleeping), 8 physical health (i.e., frequency of drinking, total alcohol intake, medication, snoring, morning tiredness, daytime fatigue, hypertension, and restless legs), 11 job characteristics (i.e., working hours per week, longest consecutive working day, transportation for commuting, exhaustion after work, strain from working, passiveness at work, work safety, increased workload, sufficient rest during work, easiness of re-employment, and risk of job-loss), and 26 mental health (i.e., wish to fly airplane, general health, pain and discomfort, satisfaction with appearance, accessibility to information, mobility, satisfaction with transportation, vulnerability to tiredness, avoidance of difficulty, distressing trivial thoughts, steadiness, tension over concerns, elevated mood, self-confidence, interest in sex, excessive or risky behavior, easiness to wake up, best time for mental activities, appetite in the morning, well-balanced meal, frequency of smoking, family and interpersonal stress, stress by relationship changes, stress by unexpected happening, imagination, and fast recovery after difficulties).

**Table 3:** Predictors of insomnia severity in shift and non-shift workers in the ML model.

Categories	Origin	Predictors of insomnia severity	Predictors in shift workers	Predictors in non-shift workers
Demographic	Consensus	Co-sleeping		
Physical Health	Consensus	Frequency of drinking	✓	
		Total alcohol intake	✓	
		Medication	✓	
	BQ	Snoring	✓	✓
		Morning tiredness	✓	✓
		Daytime fatigue	✓	
		Hypertension	✓	
Job Characteristics	Consensus	Working hours per week	✓	
		Average consecutive working days	✓	
		Longest consecutive working day	✓	
		Sideline	✓	
	MBI-GS	Exhaustion after work	✓	
		Strain from working	✓	
		Tiredness before work	✓	✓
		Passiveness at work	✓	
	Korean Occupational Stress Scale	Work safety	✓	
		Increased workload	✓	
		Sufficient rest during work		✓
		Easiness of re-employment	✓	
		Risk of job-loss	✓	
		Authoritarian work atmosphere	✓	
Mental Health	UPPS-P	Wish to fly an airplane	✓	
	WHOQOL-BREF	General Health	✓	✓
		Pain and discomfort	✓	✓
		Satisfaction with appearance	✓	
		Accessibility to information	✓	
		Mobility	✓	✓
		Satisfaction with transportation	✓	✓
	STAI-T	Vulnerability to tiredness		✓
		Avoidance of difficulty	✓	
		Distressing trivial thoughts	✓	✓
		Steadiness	✓	✓
		Tension over concerns	✓	
	MDQ	Self-confidence	✓	
		Interest in sex	✓	
		Excessive or risky behavior	✓	
	MEQ	Easiness to wake up	✓	
		Appetite in the morning	✓	
	SVS	Well-balanced meal	✓	✓
	GARS	Family and interpersonal stress	✓	
		Stress by relationship changes	✓	✓
		Stress by unexpected happening	✓	
	BFI	Imagination		✓
	BRS	Fast recovery after difficulties		✓

✓ indicates the significant predictors extracted by an ML algorithm. BQ, Berline Questionnaire; MBI-GS, Maslach Burnout Inventory-General Survey; UPPS-P, UPPS-P Impulsive Behavior Scale; WHOQOL-BREF, World Health Organization Quality of Life Brief Version; STAI-T, State–Trait Anxiety Inventory-Trait; MDQ, Mood Disorder Questionnaire; MEQ, Morningness–Eveningness Questionnaire; SVS, Stress Vulnerability Scale; GARS, Global Assessment of Recent Stress; BFI, Big Five Inventory; BRS, Brief Resilience Scale.

### Predictors of insomnia among shift workers

**Figure 1:**
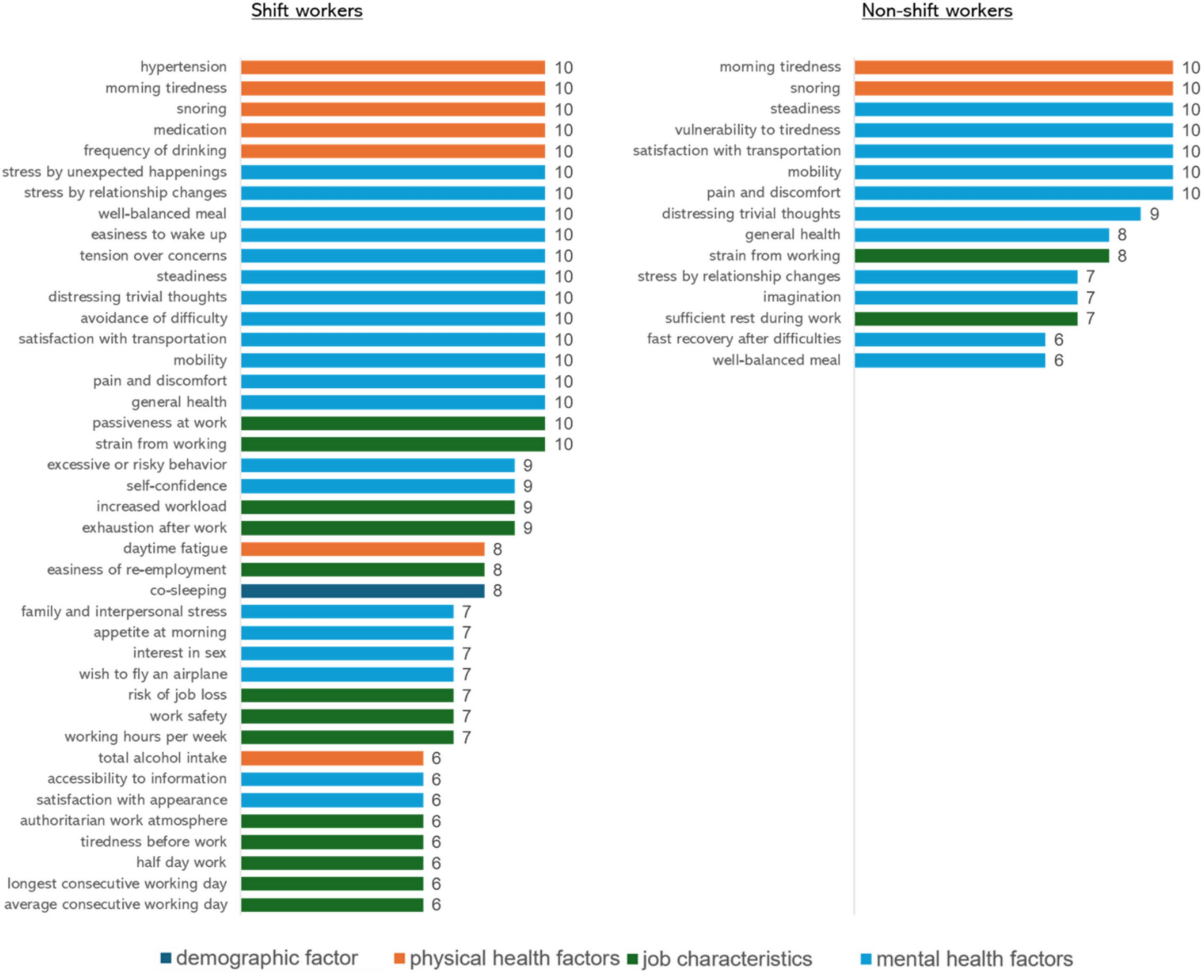
The relative importance of key predictors in machine learning models among shift and non-shift workers. In the ML model for shift workers, the predictors with the highest importance included 4 physical health factors (hypertension, morning tiredness, snoring, medication, and frequency of drinking), 12 mental health factors (stress by unexpected happenings, stress by relationship changes, well-balanced meal, easiness to wake up, tension over concerns, steadiness, distressing trivial thoughts, avoidance of difficulty, satisfaction with transportation, mobility, pain and discomfort, and general health), and 2 job characteristics (passiveness at work, strain from working). 7 predictors—2 physical health factors (morning tiredness and snoring) and 5 mental health factors (steadiness, vulnerability to tiredness, satisfaction with transportation, mobility, and pain and discomfort)—were ranked as the most important predictors in the ML model for non-shift workers.

Among the 281 input variables, the ML algorithm selected 41 variables to predict insomnia among shift workers: 1 demographic factor, 7 physical health factors, 13 job characteristics, and 20 mental health factors. Figure 1 illustrates the relative importance of key predictors in the ML model among shift workers. The relative importance index represents the frequency with which each predictor was selected during 10-hold cross-validation. A higher value indicates that the feature was chosen more frequently, reflecting its relative importance in the model. The features with the highest relative importance index, selected in all 10 iterations of the 10-fold cross-validation, included 4 physical health factors (hypertension, morning tiredness, snoring, medication, and frequency of drinking), 12 mental health factors (stress by unexpected happenings, stress by relationship changes, well-balanced meal, easiness to wake up, tension over concerns, steadiness, distressing trivial thoughts, avoidance of difficulty, satisfaction with transportation, mobility, pain and discomfort, and general health), and 2 job characteristics (passiveness at work, strain from working). Following these, 2 mental health factors (excessive or risky behavior), and 2 job characteristics (increased workload, exhaustion after work) were selected in 9 out of 10 iterations, making them the second most important predictors. Next, 1 physical health factor (daytime fatigue), 1 job characteristic (easiness of re-employment), and 1 demographic factor (co-sleeping) were chosen in 8 iterations, ranking third in importance. Additionally, 4 mental health factors (family and interpersonal stress, appetite in the morning, interest in sex, wish to fly an airplane) and 3 job characteristics (risk of job loss, work safety, and working hours per week) were selected 7 times. Lastly, 1 physical health factor (total alcohol intake), 2 mental health factors (accessibility to information, satisfaction with appearance), and 5 job characteristics (authoritarian work atmosphere, tiredness before work, half day work, longest consecutive working day, and average consecutive working day) were chosen in 6 iterations, making them the fifth most important predictors. Detailed explanations about the selected predictors are presented in Supplementary Table 2.

### Predictors for insomnia among non-shift workers

Of the 281 input variables, the ML algorithm selected the following 15 variables for predicting insomnia among non-shift workers: 2 physical health factors, 2 job characteristics, and 11 mental health factors. Figure 1 illustrates the relative importance of key predictors in the ML model among non-shift workers. The features with the highest relative importance index, selected in all 10 iterations of the 10-fold cross-validation, included 2 physical health factors (morning tiredness and snoring) and 5 mental health factors (steadiness, vulnerability to tiredness, satisfaction with transportation, mobility, and pain and discomfort). Following these, 1 mental health factor (pain and discomfort) ranked as the second most important predictor. Additionally, 1 mental health (general health) and 1 job characteristic (strain from working) were selected in 8 iterations. Next, 2 job characteristics (stress by relationship changes, imagination) and 1 job characteristic (sufficient rest during work) were chosen in 7 out of 10 iterations. Lastly, 2 mental health factors (fast recovery after difficulties and well-balanced meal) were selected in 6 iterations. Detailed explanations about the selected predictors are presented in Supplementary Table 2.

### Interaction between predictors and shift work on insomnia

**Figure 2:**
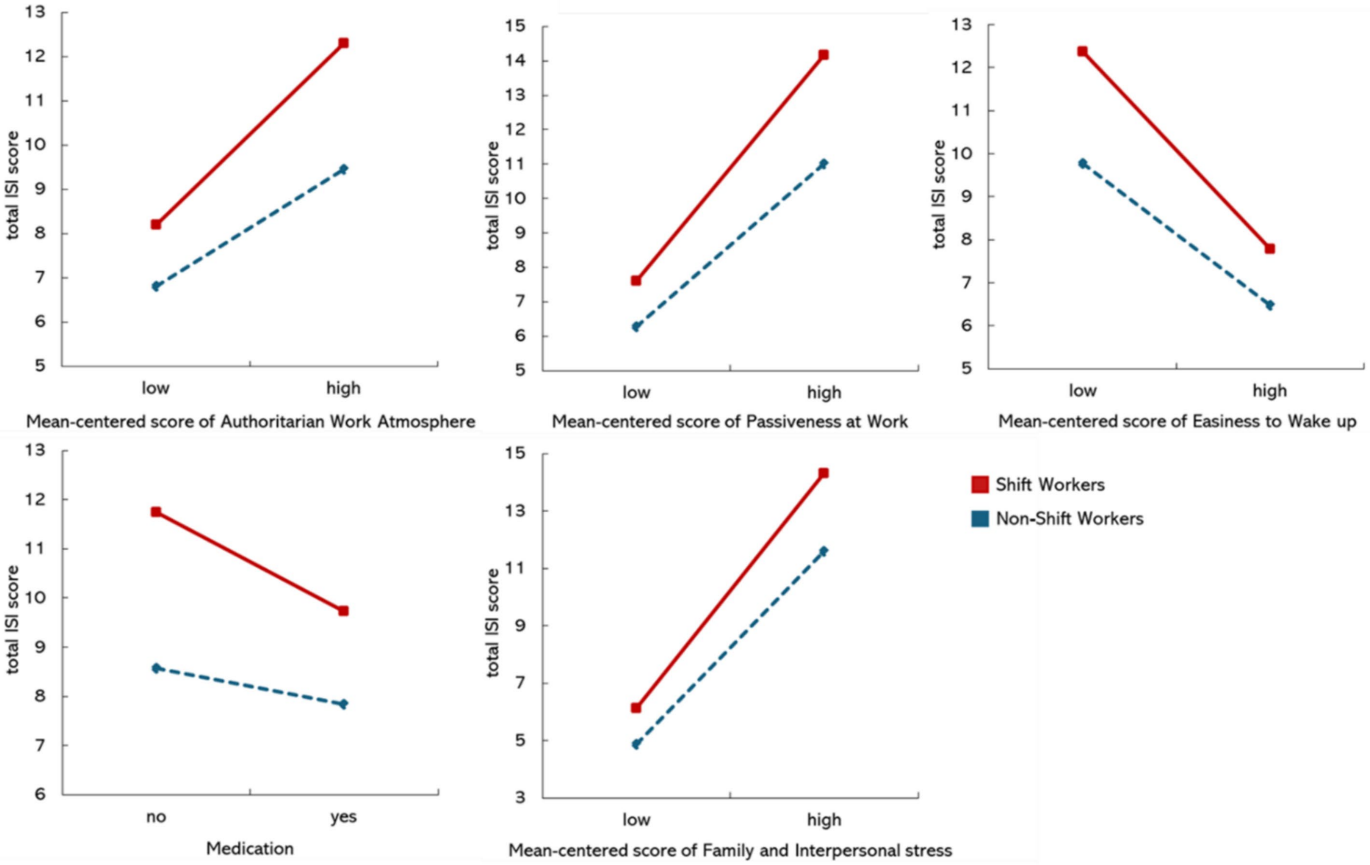
Interaction effects between five significant predictors and shift/non-shift workers in predicting insomnia severity. Five variables with substantial interaction effects with a shift work schedule in predicting the ISI scores: passiveness at work (B = 0·454, SE = 0·159, *p* = 0·004), authoritarian work atmosphere (B = 0.487, SE = 0.195, *p* = 0.013), easiness to wake up (B = −0.430, SE = 0.178, *p* = 0.016), family and interpersonal stress (B = 0.165, SE = 0.071, *p* = 0.021), and medication (B = −1.278, SE = 0.402, *p* = 0.001). The slope of the shift worker group was steeper than that of the non-shift worker group, indicating stronger interaction effects in all five variables.

The interactions between predictors and shift work on insomnia were assessed for 34 variables predicting only the insomnia of shift workers (30 variables) or non-shift workers (4 variables). Among these 34 variables, only five had substantial interaction effects with a shift work schedule in predicting ISI scores, while also exhibiting a significant correlation with ISI scores in both shift and non-shift workers: passiveness at work (B = 0.454, *p* = 0.004), authoritarian work atmosphere (B = 0.487, *p* = 0.013), easiness to wake up (B = −0.430, *p* = 0.016), family and interpersonal stress (B = 0.165, *p* = 0.021), and medication (B = −1.278, *p* = 0.001). Figure 2 shows the moderation of five variables. Furthermore, no significant interaction effects of shift work on insomnia in other variables were observed.

### Performance of machine learning model

**Figure 3:**
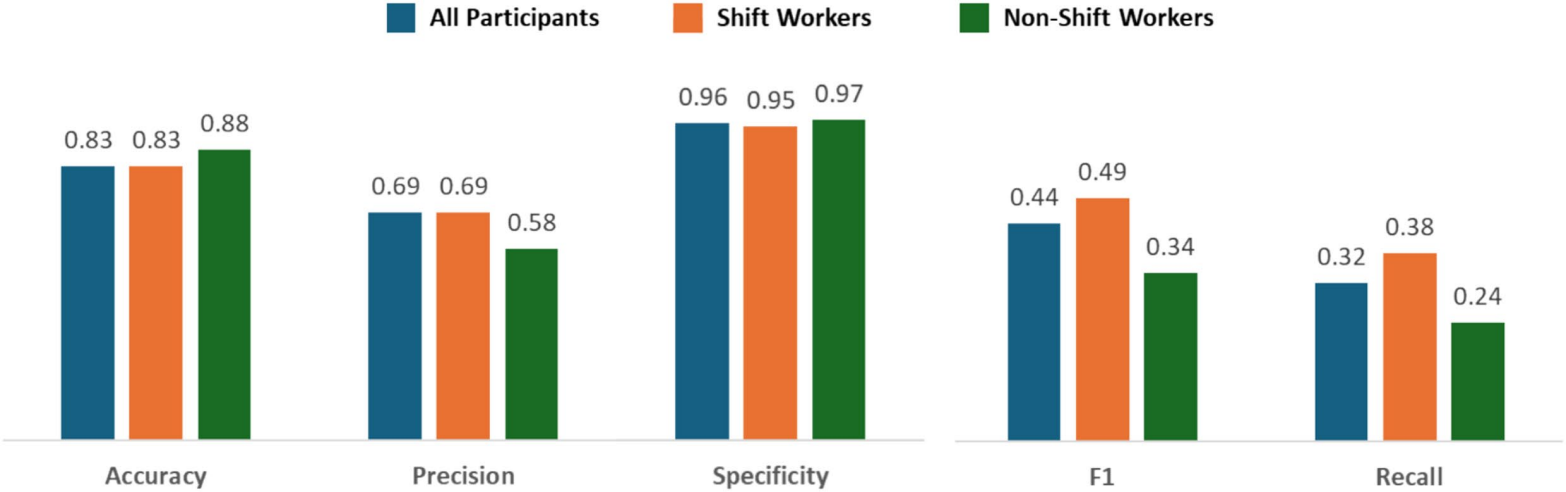
Results of performance metrics of prediction model among all participants, shift workers, and non-shift workers. The performance metrics resulting from the machine learning model showed that all models achieved high accuracy and specificity. The prediction model of shift workers showed relatively low F1 scores and recall indicating more balance in its predictions.

To evaluate the prediction performance, we used five metrics as performance indices: accuracy, F1, precision, recall, and specificity. As shown in Figure 1, the accuracy, F1, precision, recall, and specificity of the prediction model among all participants were 0.83, 0.44, 0.69, 0.32, and 0.96. The accuracy, F1, precision, recall, and specificity of the prediction model among shift workers were 0.83, 0.49, 0.69, 0.38, and 0.95. The accuracy, F1, precision, recall, and specificity of the prediction model among non-shift workers were 0.88, 0.34, 0.58, 0.24, and 0.97. Although low recall and F1 scores were suggested, all three models demonstrate a combination of high accuracy and specificity. The prediction model of shift workers showed relatively better F1 scores and recall, indicating the model is more balanced in its predictions than others (Figure 3).

## Discussion

This study aims to identify key predictors and develop an ML-based prediction model for the severity of insomnia, especially in shift workers. The ML model included a large number of diverse data, such as demographic information, job characteristics, and responses from questionnaires about physical and mental health. The application of ML techniques enabled the identification of key predictors of insomnia that may differ between shift workers and non-shift workers. By examining 281 potential items, 41 key predictors among shift workers and 15 key predictors among non-shift workers were obtained. Moreover, the distinctive predictors of insomnia specific to shift workers were identified. By leveraging ML to streamline variables, our study emphasizes the effectiveness of reducing complexity. This ML algorithm would enable a more efficient selection of key predictors of insomnia among shift workers, as well as the provision of clinical insights for the management of SWD.

The prediction model of insomnia severity in shift workers included 41 predictors among the following categories: mental health (20 predictors) followed by job characteristics (13 predictors), physical health (7 predictors), and demographics (1 predictor). The key predictors included items related to anxiety/avoidance, impulsivity, somatic complaints, and interpersonal conflicts which align with our expectations. Although the predictor such as the item ‘wish to fly an airplane,’ seems to be unrelated to insomnia, such an item can represent impulsivity which is linked to the hyperarousal model of insomnia. Occupational factors specific to shift workers play an important role in predicting insomnia as expected. These key predictors derived from 281 potential factors can be interpreted as the most efficient screening factors to detect severe insomnia in shift workers.

In line with a traditional insomnia model, items concerning anxiety/avoidance emerged as significant predictors for insomnia among shift workers. The integrative and well-established hyper-arousal model of insomnia (14) explains that heightened anxiety levels lead to cognitive arousal (i.e., difficulties in thought control and intrusive thoughts.). Excessive worry and fear over sleep make individuals much more anxious and aroused, resulting in sleep disturbance (16). These hyperaroused individuals give more attention to sleep-related stimuli and make efforts to fall asleep resulting in insomnia (15). Similarly, impulsivity may be related to difficulties in thought control (60), leading to intrusive thoughts that disturb sleep. Although no items directly assessing the previous history of the medical condition were included, items assessing complaints about physical health were identified as key predictors. Moreover, complaints about their physical health might be closely related to health anxiety. Health anxiety observed in somatic symptom disorder showed a high prevalence of insomnia, which shows a strong correlation between health anxiety and insomnia (61). Interpersonal conflicts can also contribute to insomnia severity due to increased arousal and rumination before sleep (62).

Occupational factors, such as excessive workload, burnout symptoms, job security, working atmosphere, and work autonomy, were identified as key predictors of insomnia severity in shift workers. The larger number of key factors among job characteristics emphasizes the greater impact of occupational factors on shift workers than non-shift workers; therefore, improving occupational factors may substantially mitigate insomnia severity among shift workers. Upon a closer examination of each factor, an excessive workload might directly reduce the time to rest, causing sleep deprivation. Burnout symptoms, especially work-related fatigue, might lower individuals’ tolerance to perceiving events as stressful and trigger sleep disturbance during stressful events (63). Job security, which entails a stable environment and guaranteed employment, emerges as a significant stressor in the workplace, with its loss correlating with adverse mental health effects, such as anxiety and sleep disturbances (64, 65). Furthermore, work-related factors, such as the work atmosphere and autonomy, can potentially alleviate the adverse effects of shift work on insomnia severity.

In the physical health category, it was expected that daytime fatigue and hypertension were included as the key predictors, since hypertension, an indicator of hyperarousal in insomnia, is based on physiological changes (66, 67). Given the prevalence of alcohol dependence in shift workers (68), severe insomniac shift workers tend to use alcohol to alleviate tension. Snoring and morning tiredness and core sleep-disturbing medical conditions predicted severe insomnia in both shift and non-shift workers.

Co-sleeping was the only demographic predictor for insomnia in shift workers. Despite previous uncertainties about the correlation between chronic insomnia disorder and bed-sharing (69), the unique features of shift work lead to different results. Frequent nocturnal awakening can be transmitted between co-sleeping partners (70, 71), suggesting that unstable or reversed day and night work patterns may amplify sleep problems for both shift workers and their partners.

Five predictors, including passiveness at work, authoritarian work atmosphere, family and interpersonal stress, ease of waking up, and medication, were identified to significantly vary in their impacts on insomnia severity depending on their work schedules. All these predictors showed stronger associations with shift workers compared with non-shift workers, which provides insights into which factors exacerbate or alleviate insomnia severity specifically among shift workers. First, shift workers are more susceptible to insomnia when they are unable to actively demonstrate their opinions at work or working under an authoritative boss. This suggests that their work environment influences the severity of insomnia. Given the nature of shift work, workers need to communicate more with their supervisors to adjust their work schedules flexibly to prevent insomnia, but a strict and authoritative workplace atmosphere makes this difficult. Unlike non-shift workers, the limited freedom to adjust their schedules is one of the distinctive stressors that shift workers experience. Second, greater interpersonal stress exacerbates insomnia in both shift and non-shift workers, but its negative effect is more evident among shift workers. Our findings also suggest that shift workers tend to have higher levels of family and interpersonal stress, indicating that support from their social networks is important in alleviating insomnia severity. Third, shift workers who have difficulties waking up tend to experience more severe insomnia, and this negative effect is stronger compared with that in non-shift workers. It can be concluded the insomnia symptoms of shift workers are not closely related to terminal insomnia, where the sleeper wakes up earlier than desired. In other words, insomnia in shift workers might be more related to delayed circadian rhythms characterized by sleep-onset insomnia when attempting sleep at conventional times. Lastly, the shift workers have experienced a significant decrease in insomnia severity when taking medication. Although medication information was not reported, individuals with insomnia were more likely to use sleep-aiding medicine. This suggests that the effects of sleep aids may be more evident among shift workers. Alternatively, it could be implied that an irregular working schedule prevents shift workers from consistently visiting hospitals or taking medicines, leading to untreated sleep problems.

All of our ML models predict insomnia in shift and non-shift workers, and all models showed high accuracy, specificity, and precision but low recall and F1 scores. High accuracy indicates that the model correctly predicts the severity of insomnia for a large proportion of participants, while high specificity indicates the model’s ability to effectively identify individuals with lower insomnia severity, minimizing false positives for severe insomnia. On the other hand, low recall suggests that the model fails to adequately detect shift workers with severe insomnia, misclassifying some individuals as having lower severity. Additionally, a low F1 score with high precision but low recall implies that the model effectively detects mild insomnia cases but is likely to miss severe insomnia cases. Overall, the model can sensitively detect individuals with insomnia but may underestimate those with severe insomnia in the real world. Despite its potential for early screening, its limited recall and F1 scores suggest that further studies are needed before it can be applied in clinical settings. Our findings can serve as a basis for generating hypotheses to guide future research aimed at improving model accuracy and identifying more reliable predictors of severe insomnia in shift workers.

Our findings highlight the importance of occupational factors, particularly how shift workers subjectively assess their work environment. While several studies have examined the impact of individual vulnerabilities—such as neuroticism (72), morningness-eveningness chronotypes (73, 74), or the night shift schedule (75)—less attention has been given to the influence of organizational climates. The higher ratings on items like “passive at work” (I am passive when it comes to doing the tasks assigned to me) and “authoritarian work atmosphere” (The atmosphere at my workplace is authoritarian and hierarchical) were associated with more severe insomnia in shift workers compared to non-shift workers. Job stress such as discomfort in occupational climate was significantly associated with insomnia (76). Shift workers experienced more burnout than non-shift workers (77), and job strain was associated with difficulties in initiating sleep in shift workers (78). Reduced autonomy in shift work may prevent workers from aligning their tasks and rest with their biological rhythm, increasing the risk of circadian rhythm disorders and insomnia. Moreover, the less perceived organizational support, the more severe insomnia among shift-working nurses (79). These findings suggest that organizational factors may play a role in the severity of insomnia among shift workers. Promoting a more horizontal organizational culture, with greater flexibility in scheduling and workload distribution, could potentially reduce feelings of passivity and improve their sense of control. This, in turn, may have a positive impact on sleep outcomes. Additionally, fostering a culture that supports assertiveness and open communication could help mitigate the negative effects of an authoritarian work environment. While these changes hold promise, further research is needed to explore their effectiveness and determine how they might contribute to improving sleep quality and overall well-being among shift workers.

There are several limitations in our study. Firstly, given the exploratory nature of this study based on cross-sectional data, the associations identified in the predictive model are correlational rather than causal. Further investigation using longitudinal or experimental designs is necessary to establish causal relationships and improve generalizability. Second, while the hyperarousal model of insomnia aligns with the results of our ML model, no variables directly measuring hyperarousal were included. Incorporating variables that capture the multifaceted aspects of hyperarousal as input variables could improve the model’s performance. Third, voluntary recruitment may have led to selection bias by excluding individuals with severe psychopathology. Fourth, reliance on self-reported questionnaires may have compromised the accuracy of the participants in reporting their experiences. Fifth, the lack of detailed medication information limited the precise interpretation of the findings. Lastly, because all available data were used to improve modeling accuracy, independent validation data are needed in future studies.

## Conclusion

The current study explored an ML-based approach to identifying key predictors and developing a predictive model for insomnia in shift workers. By exploring a wide range of potential predictors, including demographic information, job characteristics, and physical and mental health, our study derived meaningful insights into insomnia in shift workers. Some key predictors identified through our ML model included anxiety-related factors consistent with the traditional hyperarousal model of insomnia. Notably, job-specific features such as work culture and interpersonal relationships, distinguished insomnia among shift workers from that among non-shift workers. Given the exploratory nature of this study, longitudinal research and independent validation are necessary to establish the relevance and utility of these predictors in clinical and occupational settings.

## Data Availability

The original contributions presented in the study are included in the article/Supplementary material, further inquiries can be directed to the corresponding author.
