# Supplementary material for: Exploring predictors of insomnia severity in shift workers using machine learning model
Source: Front Public Health. 2025 Mar 14;13:1494583. doi: 10.3389/fpubh.2025.1494583 (PMC11949791; doi:10.3389/fpubh.2025.1494583)
Supplement: Supplementary file 1 [file Table_1.DOCX]

**Supplementary Table 1. Detailed explanations about potential predictors.**

| **Variables** | **Explanations** |
| --- | --- |
| Gender (1 item) | What is your gender? |
| Age (1 item) | How old are you? |
| Education (1 item) | What is your highest level of education? |
| Family (4 items) | What is your marital status?  Do you currently live with any family members?  How many family members do you live with?  Do you share a bed or blanket with someone when you sleep? |
| Work schedule (25 items) | Which of the following best matches your work arrangement?  On average, how many days per week do you work in a non-regular schedule? On average, how many days do you work consecutively without any rest days? What is the longest period you work consecutively without any rest days? On average, how many times do you work consecutively with the same schedule? _(1) Consecutive regular schedule workdays _(2) Consecutive afternoon/evening workdays _(3) Consecutive night/early morning workdays _(4) Other On average, how many days do you work consecutively at night without any rest days? What is the longest period you work consecutively at night without any rest days? On average, how many consecutive rest days do you have? What is the longest period of consecutive rest days you have? How many weekends with rest days do you have per month on average? In the past year, have you had a continuous period of more than one week with rest days? Which of the following work schedules have you experienced? _(1) Regular schedule work _(2) Afternoon or evening work _(3) Night or early morning work _(4) Half-day work outside regular schedule _(5) Full-day work outside regular schedule _(6) Other schedules  Please specify the start times of your regular working hours.  Please specify the end times of your regular working hours.  Please specify the start times of your other work schedules.  Please specify the end times of your other work schedules.  How many weekends with rest days do you have per month on average?  How far in advance do you typically know your work schedule (shift roster) outside of your regular working hours? |
| Time-zone shift (1 item) | Does your job involve working across multiple time zones, causing you to experience jet lag repeatedly? |
| Working period (3 items) | How long have you been working in total, including your current job?  How long have you been working in your current shift work/irregular work schedule?  How long in total have you worked in shift work/irregular work schedules, including any past positions? |
| Working hours per week  (1 item) | On average, how many hours do you work per week? |
| Sideline (1 item) | Do you have any side jobs or additional sources of income besides your main occupation? |
| Commute (3 items) | Please indicate the average time it takes for you to commute to work. Please indicate the average time it takes for you to commute home.  What mode of transportation do you use most frequently for commuting? |
| Self-reported workload  (4 items) | Compared to others in similar occupations, how would you rate the physical workload burden? Compared to others in similar occupations, how would you rate the mental workload burden? Compared to others in similar occupations, how would you rate the time pressure burden? Compared to others in similar occupations, how would you rate the emotional stress burden? |
| Height/weight (3 items) | What is your height  What is your weight?  Calculated BMI |
| Alcohol/smoking/coffee  (5 items) | How often do you drink alcohol?  What type of alcohol do you primarily enjoy?  How much alcohol do you typically consume in total when you drink?  Which of the following best describes your current smoking status?  How much coffee do you consume per day? |
| Current/past medical disease  (2 items) | Do you currently have any medical or physical conditions?  Have you had any medical or physical conditions in the past? |
| Medication (1 item) | Are you currently taking any medications? |
| Family history of sleep disorder (1 item) | Has anyone in your family (parents, siblings, children) experienced sleep disorders, either currently or in the past? |
| Eating/diet (4 items) | Do you regularly eat breakfast?  Do you regularly have three meals a day?  How often do you tend to overeat?  In the past year, have you made any efforts to control your weight? |
| Exercise (3 items) | How often do you engage in intense exercise that significantly increases your breathlessness for more than 20 minutes a day?  How often do you engage in moderate-intensity exercise that slightly increases your breathlessness for more than 30 minutes a day?  How often do you walk for a total of more than 30 minutes a day, including at least one instance of walking for 10 minutes or more? |

The items extracted from the questionnaires were not included.

**Supplementary Table 2. Detailed explanations about selected predictors in prediction models.**

| **Variables** | **Explanations**^a^ |
| --- | --- |
| Co-sleeping | Do you share a bed or blanket with someone when you sleep? |
| Frequency of drinking | How often do you drink alcohol? |
| Total alcohol intake | How much alcohol do you typically consume in total when you drink? |
| Medication | Are you currently taking any medications? |
| Snoring | Do you snore? |
| Morning tiredness | How often do you feel tired or fatigued after you sleep> |
| Daytime fatigue | During your waking time, do you feel tired, fatigued or not up to par? |
| Hypertension | Do you have high blood pressure? |
| Working hours per week | On average, how many hours do you work per week? |
| Average consecutive working days | On average, how many days do you work consecutively without any rest days? |
| Longest consecutive working day | What is the longest period you work consecutively without any rest days? |
| Sideline | Do you have any side jobs or additional sources of income besides your main occupation? |
| Exhaustion after work | I feel completely exhausted when I leave work. |
| Strain from working | I feel tense from working all day. |
| Tiredness before work | I feel tired at the thought of getting up and going to work in the morning. |
| Passiveness at work | I am passive when it comes to doing the tasks assigned to me. |
| Work safety | My job is risky, and there is a possibility of an accident. |
| Increased workload | The workload has significantly increased. |
| Sufficient rest during work | I am given enough breaks during work. |
| Easiness of re-employment | Even if I quit my current job, I can easily find a job at the same level. |
| Risk of job-loss | There is a possibility that I could lose my current job within the next two years. |
| Authoritarian work atmosphere | The atmosphere at my workplace is authoritarian and hierarchical. |
| Wish to fly an airplane | I want to learn how to fly a plane. |
| General Health | How satisfied are you with your health? |
| Pain and discomfort | How much do you feel that physical pain interferes with the things you need to do? |
| Satisfaction with appearance | Are you satisfied with your physical appearance? |
| Accessibility to information | How easily can you access the information you need in your daily life? |
| Mobility | How well are you able to move around? |
| Satisfaction with transportation | How satisfied are you with the transportation you use? |
| Vulnerability to tiredness | I get tired easily. |
| Avoidance of difficulty | I try to avoid crises or difficulties. |
| Distressing trivial thoughts | Minor thoughts bother me. |
| Steadiness | I am a diligent person. |
| Tension over concerns | When I think about my current worries or concerns, I feel tense and unsure of what to do. |
| Self-confidence | There have been times when I felt more confident than usual. |
| Interest in sex | There have been times when I felt more interested in sexual activities than usual. |
| Excessive or risky behavior | There have been times when I acted in a way that wasn't typical for me, or when others might have thought my behavior was excessive, foolish, or risky. |
| Easiness to wake up | If you sleep in a suitable environment, is it easy for you to wake up in the morning? |
| Appetite at morning | How is your appetite during the first 30 minutes after waking up in the morning? |
| Well-balanced meal | I have at least one warm, balanced, and nutritious meal a day. |
| Family and interpersonal stress | How much pressure do you feel in your interpersonal relationships? |
| Stress by relationship changes | How much pressure do you feel due to changes in your interpersonal relationships? |
| Stress by unexpected happening | How much pressure do you feel due to events that are not part of your daily routine? |
| Imagination | I think of myself as having a rich imagination. |
| Fast recovery after difficulties | I tend to recover quickly after going through tough times. |

^a^Descriptions of some variables are adapted from the original questionnaire, with some modifications made to respect copyright.
